# Black Walnut (*Juglans nigra*) Extracts Inhibit Proinflammatory Cytokine Production From Lipopolysaccharide-Stimulated Human Promonocytic Cell Line U-937

**DOI:** 10.3389/fphar.2019.01059

**Published:** 2019-09-19

**Authors:** Khanh-Van Ho, Kathy L. Schreiber, Danh C. Vu, Susan M. Rottinghaus, Daniel E. Jackson, Charles R. Brown, Zhentian Lei, Lloyd W. Sumner, Mark V. Coggeshall, Chung-Ho Lin

**Affiliations:** ^1^Center for Agroforestry, School of Natural Resources, University of Missouri, Columbia, MO, United States; ^2^Department of Food Technology, Can Tho University, Can Tho, Vietnam; ^3^Cell and Immunobiology Core, University of Missouri, Columbia, MO, United States; ^4^Department of Veterinary Pathobiology, University of Missouri, Columbia, MO, United States; ^5^Metabolomics Center, University of Missouri, Columbia, MO, United States; ^6^Department of Biochemistry, Bond Life Sciences Center, University of Missouri, Columbia, MO, United States; ^7^United States Northern Research Station, USDA-Forest Service, West Lafayette, IN, United States

**Keywords:** black walnut, *Juglans nigra*, metabolomic profiling, cytokine suppression, potential anti-inflammatory compounds

## Abstract

Black walnut (*Juglans nigra* L.) is an excellent source of health-promoting compounds. Consumption of black walnuts has been linked to many health benefits (e.g., anti-inflammatory) stemming from its phytochemical composition and medicinal properties, but these effects have not been systematically studied or characterized. In this study, potential anti-inflammatory compounds found in kernel extracts of 10 black walnut cultivars were putatively identified using a metabolomic profiling analysis, revealing differences in potential anti-inflammatory capacities among examined cultivars. Five cultivars were examined for activities in the human promonocytic cell line U-937 by evaluating the effects of the extracts on the expression of six human inflammatory cytokines/chemokines using a bead-based, flow cytometric multiplex assay. The methanolic extracts of these cultivars were added at four concentrations (0.1, 0.3, 1, and 10 mg/ml) either before and after the addition of lipopolysaccharide (LPS) to human U-937 cells to examine their effect on cytokine production. Results from cytotoxicity and viability assays revealed that the kernel extracts had no toxic effect on the U-937 cells. Of the 13 cytokines [interleukin (IL)-1β, tumor necrosis factor alpha (TNF-α), monocyte chemoattractant protein (MCP)-1, IL-6, IL-8, IL-10, IL-12, IL-17, IL-18, IL-23, IL-33, interferon (IFN)-α, IFN-γ] measured, only six were detected under the culture conditions. The production of the six detected cytokines by phorbol 12-myristate 13-acetate (PMA)-differentiated, LPS-stimulated U-937 was significantly inhibited by the kernel extracts from two cultivars Surprise and Sparrow when the extracts were added before the addition of LPS. Other cultivars (Daniel, Mystry, and Sparks) showed weak or no significant effects on cytokine production. In contrast, no inhibitory effect was observed on the production of cytokines by PMA-differentiated, LPS-stimulated U-937 when the kernel extracts were added after the addition of LPS. The findings suggest that the extracts from certain black walnut cultivars, such as Sparrow and Surprise, are promising biological candidates for potentially decreasing the severity of inflammatory disease.

## Introduction

Inflammation is a complex pathophysiological response of the immune system in response to infections of harmful stimuli or tissue damage (Medzhitov, 2010). An uncontrolled inflammatory response might result in development of a variety of chronic inflammatory diseases such as rheumatoid arthritis (RA). RA is the most common inflammatory arthritis affecting ∼1% of population worldwide (Firestein, 2003). This inflammatory disease is associated with articular inflammation and synovial joint damage that can result in disability and increased mortality. In synovial tissues, several cytokines are released and are functionally active in mediating immune responses associated with the pathogenesis of RA (McInnes and Schett, 2007). Cytokines are therapeutic targets for the treatment of patients with RA [tumor necrosis factor (TNF)] and in RA clinical trials [e.g., interleukin (IL)-1, IL-6, IL-27] (McInnes et al., 2016).

Black walnut (*Juglans nigra* L.) is one of the most economically valuable hardwood species in the United States (Randolph et al., 2013). Native Americans traditionally valued kernels of black walnut as a food source and utilized leaves for medical purposes to treat diarrhea, bilious, and cramp colic (Nolan and Robbins, 1999). A recent report indicated that the kernels of black walnut contain a wealth of phytochemical substances (Vu et al., 2018), and many of these phenolic compounds are associated with a variety of biological functions such as antioxidant, antimicrobial, and anti-inflammatory properties. Our previous study demonstrated antibacterial activities of the kernels from black walnut against a Gram-positive bacterium (*Staphylococcus aureus* RN6390), and its antibacterial compounds (e.g., glansreginin A, azelaic acid) were successfully identified (Ho et al., 2018).

Anti-inflammatory capacities of another common *Juglans* species, English walnut (*Juglans regia* L.) have been well established *in vitro* and *in vivo*. Willis et al. (2010) reported that kernel extracts of English walnut reduced production of TNF-α and nitric oxide synthase induced by BV-2 murine microglial cells stimulated with lipopolysaccharide (LPS) when the cells were pretreated with the extract before stimulation with LPS. Papoutsi et al. (2008) also documented that extracts of English walnut decreased expression of TNF-α-induced endothelial vascular cell adhesion molecule (VCAM)-1 and intracellular adhesion molecule (ICAM)-1 expression in human aortic endothelial cells. *In vivo*, consumption of English walnut was associated with multiple health benefits with respect to alleviating inflammation and oxidative stress and improving vascular function in both animals and human clinical trials (Ros, 2015). In fact, the consumption of English walnut was associated with a lowered risk of type 2 diabetes (Pan et al., 2013) and a reduction in the incidence of major cardiovascular events (Estruch et al., 2013) in two randomized clinical trials of 137,956 women aged 35–77 over 10 years and of 7,447 participants aged 55–80 (47% were men), respectively. Anti-inflammatory functions of English walnut are likely associated with its phytochemical compounds. Remarkably, English walnut and black walnut share a similar phytochemical profile, revealing potential anti-inflammatory capacities of black walnut extracts. In fact, 16 phenolic compounds characterized in English walnut are also found in black walnut (Vu et al., 2018). Nonetheless, anti-inflammatory properties of black walnut have never been characterized yet. Exploring the anti-inflammatory properties of black walnut might point to novel uses of black walnut and its by-products, promoting development of new biological agents for the prevention or even treatment of inflammation as well as increasing the value of black walnuts by identifying new applications and health benefits.

In the present study, we first examined and compared effects of kernel extracts from 10 black walnut cultivars *via* a global metabolomic profiling approach. We then characterized possible anti-inflammatory properties in kernel extracts of five selective cultivars by evaluating the expression of six inflammatory mediators (IL-1β, TNF-α, MCP-1, IL-6, IL-8, and IL-10) using the human promonocytic cell line U-937.

## Materials and Methods

**Black walnut cultivars.** The nuts of 10 black walnut cultivars including Daniel, Davidson, Hay, Jackson, Kwik Krop, Mystry, Schessler, Sparks, Sparrow, and Surprise were collected at the University of Missouri, Horticulture and Agroforestry Research Center (New Franklin, MO, USA). The black walnuts were hulled mechanically and hung up to dry at 24°C for 15 days. The hulled nuts were then stored at −20°C until analysis.

**Sample preparation.** The hulled nuts were cracked manually and the kernels removed from the shell and homogenized using a coffee grinder (CBG100S, Black + Decker, Beachwood, OH, USA). For metabolomic analysis, the kernels (2.5 g, 20–30 mesh) from 10 cultivars were extracted by sonication in 15 ml methanol. The methanolic extract was sonicated in a water bath (4°C) for 60 min followed by centrifugation for 10 min at 8,000 rpm. The supernatant was collected and filtered through a 0.2-µm Whatman Anotop syringe membrane filter (Sigma-Aldrich, St. Louis, MO, USA) and then injected into ultra high-performance liquid chromatography tandem mass spectrometry (UHPLC-MS). For assays, the kernels (2.5 g) from five selective cultivars (i.e., Daniel, Mystry, Sparks, Sparrow, and Surprise) were extracted by sonication in 10 ml of methanol. The extract was sonicated for 60 min followed by centrifugation for 10 min at 4,000 rpm, and the supernatant was collected. Subsequently, the supernatant was filtered through the syringe membrane filter (0.2 μm, Whatman). The aqueous extract was evaporated until dryness under a flow of nitrogen and was resuspended with 0.125 ml of dimethyl sulfoxide (DMSO) (Sigma-Aldrich, USA), and then, the resulting extract was concentrated 40 times to achieve the concentration of 10,000 mg/ml. Cytokine-modulating activities of the extract were identified using multiplex bead-based cytokine assay kits (BioLegendplex^TM^ Human Inflammation panel kits, BioLegend, San Diego, CA, USA).

**UHPLC-QTOF-MS analysis to examine metabolite profiles of black walnut.** The kernel extracts (2 µl per injection) from 10 black walnut cultivars were analyzed by UHPLC coupled to a maXis impact quadrupole time-of-flight mass spectrometer (Bruker Co., Billerica, MA, USA). Separations were achieved on a Waters Acquity UHPLC BEH C18 column (2.1 × 150 mm, 1.7 µm particle size) using a linear gradient of 95%/5–30%/70% eluents A/B (A: 0.1% formic acid and B: acetonitrile) in 30 min. Subsequently, the separation was followed by a linear wash gradient as follows: 70–95% B (30–33 min), 95% B (33–35 min), 95–5% B (35–36 min), and 5% B (37–40 min), respectively. The column temperature was kept at 60°C, and the flow rate was 0.56 ml/min. Mass spectrometry was performed in both negative and positive electrospray ionization modes with the nebulization gas pressure at 43.5 psi, dry gas of 12 l/min, dry temperature of 250°C, and a capillary voltage of 4,000 V. Mass spectral data were collected automatically from 100 to 1,500 m/z; three precursors were selected for auto MS/MS and m/z range auto-calibrated using sodium formate after data acquisition. Each cultivar was analyzed in triplicate along with methanol blank used as a control.

**Cell culture and differentiation induction.** The human monocyte cell line U-937 was purchased from American Type Culture Collection (ATCC) (CRL-1593.2, ATCC, Manassas, VA, USA). U-937 cells were cultured in complete Roswell Park Memorial Institute (RPMI) medium (RPMI 1640, ATCC) supplemented with 10% fetal bovine serum (FBS, Sigma-Aldrich) and 100 µg/ml gentamicin, and then incubated at 37°C in a 5% CO_2_ humidified atmosphere. U-937 cells were seeded at 2 × 10^5^ cells/well in 96-well plates in a 200-µl volume in the presence of 50 nM of phorbol 12-myristate 13-acetate (PMA, Sigma-Aldrich) for 48 h (Rovera et al., 1979) to induce differentiation. After washing the PMA-differentiated cells twice, fresh media was added, and the cultures were incubated for an additional 18 h at 37°C in a 5% CO_2_ humidified atmosphere before the addition of extracts. Subsequently, the cultures were pretreated with extract dilutions from five cultivars (Daniel, Mystry, Sparks, Sparrow, and Surprise) at four final concentrations (0.1, 0.3, 1, and 10 mg/ml) for 2 h before stimulation with 1 µg/ml LPS (*Escherichia coli* 0127:B8, Sigma-Aldrich). In some experiments, the PMA-differentiated cells were stimulated with LPS for 2 h before the addition of two cultivar extracts (Sparrow and Surprise). Extracts were prepared in tissue-culture-grade DMSO, and the highest concentration of DMSO in any cultivar sample was 0.1%. An immunosuppressant drug, cyclosporin A (CSA, Sigma-Aldrich), was added at a final concentration of 0.002 mg/ml in 0.02% DMSO and served as a positive control for the inhibition of cytokine secretion. Samples without CSA or cultivars were supplemented with 0.1% DMSO and served as vehicle controls. Twenty-four hours after the addition of LPS and extracts, the triplicate culture supernatants from each group were pooled, spun to remove cell debris, transferred to new tubes, and stored at −20°C until analysis. Cytokine secretion levels in LPS-stimulated, cultivar-treated cultures were compared to samples containing DMSO but not cultivars. Viability of the attached cells was determined using a 3-(4, 5-dimethylthiazol-2-yl)-2, 5-diphenyltetrazolium bromide (MTT) assay (see methods section below).

A macrophage model system was chosen to investigate the anti-inflammatory potential of black walnut extracts because macrophages are central to the inflammatory response and are active during all phases of inflammation. The U-937 cell line can be induced to differentiate with PMA, and the addition of LPS derived from Gram-negative bacterial cell walls results in the release of numerous inflammatory mediators. These cytokines include, but are not limited to, TNF-α, IL-1β, and IL-6, which in turn contribute to the recruitment and activation of other immune cells against bacterial infections (Gordon, 2002). Since cytokines such as TNF, IL-6, and IL-1β play primary roles in the pathogenesis of RA (Smolen and Aletaha, 2015), they can be used as key biomarkers of RA.

**Quantification of secretion of cytokines/chemokines by macrophages.** The LEGENDplex^TM^ human inflammation bead-based immunoassay was used to quantitate the secretion of cytokines in the U-937 culture model system in the absence or presence of black walnut extract, according to the manufacturer’s procedure. Initial experiments were performed using a predefined panel of 13 inflammatory analytes (IL-1β, TNF-α, MCP-1, IL-6, IL-8, IL-10, IL-12, IL-17, IL-18, IL-23, IL-33, IFN-α, and IFN-γ), but subsequent experiments focused on a subset of six cytokines. Data were collected on a BD LSR Fortessa™ X-20 cell analyzer (BD Biosciences, San Jose, CA, USA) using instrument settings recommended by BioLegend. A cytokine standard curve was included in each experiment, and cytokine levels were calculated from a five-parameter logistic curve using the software provided in the BioLegend kit. Triplicates were collected at each cultivar concentration in multiple experiments.

**Effects of black walnut extracts on cell viability.** The MTT assays were performed to evaluate the effect of black walnut extracts on cytotoxicity and/or cell loss at the time of supernatant collection. A colorimetric cell viability assay (CGD1-1KT, Sigma-Aldrich) using MTT as the substrate (Mosmann, 1983) was carried out after collection of the supernatants. The ability of the cells to convert MTT to formazan crystals indicates mitochondrial activity and cell viability. Dulbecco’s modified eagle medium high-glucose phenol red free media (Fisher Scientific, Pittsburgh, PA, USA) containing 1% FBS and MTT was added to each well, and the plates were incubated for 3 h at 37°C until precipitates were observed. An acidified isopropanol solvent was then added to dissolve the formazan crystals, and the samples were pipetted several times to completely dissolve the crystals. Absorbance was measured within 30 min after solvent addition using a BioTek ELx808 microplate reader (BioTek, Winooski, Vermont, USA). Formazan crystals were detected at a wavelength of 570 nm, and background absorbance was measured at 630 nm.

### Statistical Analysis

For the metabolic analyses, the original UHPLC-MS data were converted into a format (*.cdf) that is compatible with XCMS Online (http://xcmsonline.scripps.edu) (Tautenhahn et al., 2012), and they were processed using the XCMS Online algorithms. This web-based tool for metabolomics data allows converting data file for peak detection, peak grouping, retention time correction, and alignment. Pairwise analyses between each black walnut cultivar and the control (methanol) were performed to identify single ion (m/z) features that were significantly different at *p* < 0.005 and intensity ≥10,000 across the chromatographic time domain. The metabolites of the significant ion features were putatively identified based on the accurate mass of the molecular ions, referenced to METLIN metabolite mass spectral database containing over 1 million molecules (http://metlin.scripps.edu) (Guijas et al., 2018). Metabolites that have been reported to possess anti-inflammatory activities based on a literature search (**Supplementary Table 1**) were selected to initially examine profiles of black walnut cultivars and to identify potential anti-inflammatory compounds in each cultivar. Multivariate analyses such as partial least squares discriminant analysis (PLS-DA) and heat map were performed using MetaboAnalyst (Chong et al., 2018) to reveal differences in metabolic profiles among black walnut cultivars.

Relative cell viability was measured with the MTT assay according to the manufacturer’s protocol. The amount of MTT conversion was determined by subtracting the background absorbance (630 nm) from the absorbance of formazan crystals at 570 nm (A_570 nm_ − A_630 nm_ = specific MTT absorbance). Cell viability was expressed relatively to the PMA-differentiated, LPS-stimulated sample (control sample) in the absence of black walnut cultivars. The percentage of cell viability (%) was calculated by dividing the specific MTT absorbance of the treated sample by the specific MTT absorbance of the control sample and multiplying by 100. The control sample was set at 100%.

Cytokine concentrations were determined in the multiplex bead-based assay by extrapolating the concentration from a five-parameter logistic standard curve using curve-fitting software. The absolute concentrations of each cytokine from treated samples were then compared to the control samples, as described above for the MTT assay. The relative percentage (%) was obtained by dividing the cytokine concentration of the treated sample by the cytokine concentration of the control sample and multiplying by 100. The PMA-differentiated, LPS-stimulated sample served as the control sample and was set at 100%.

The data were analyzed as a randomized complete block design using PROC MIXED in SAS 9.4 (SAS Institute, Cary, NC). The black walnut extract was the fixed effect, and replication was the random variable. Differences between extracts were determined using Fisher’s least significant difference at *p* < 0.01.

## Results

**Figure 1:**
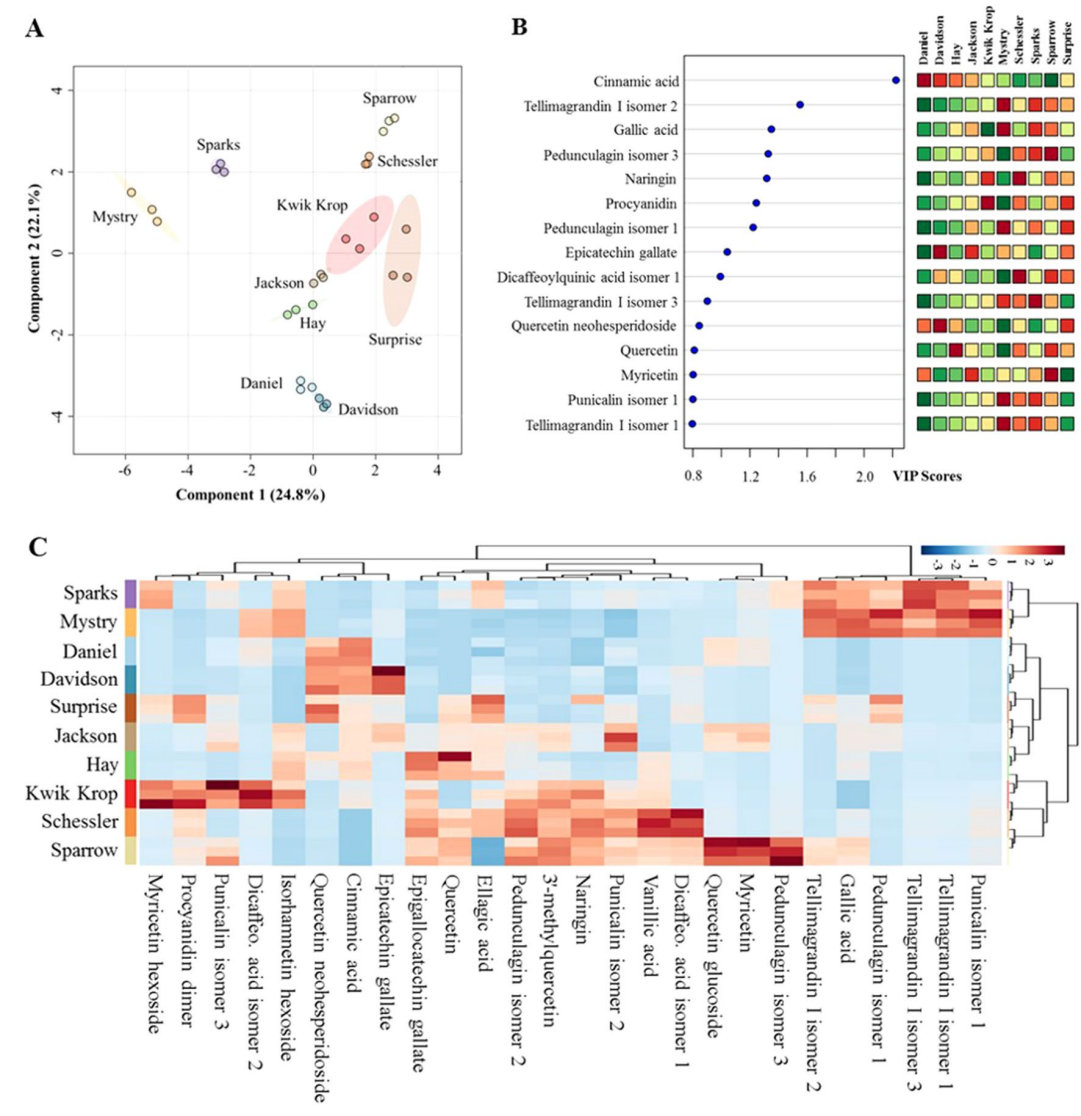
Differences in metabolic profiles of black walnut cultivars. **(A)** Partial least squares discriminant analysis (PLS-DA). **(B)** Variable importance in projection (VIP) and **(C)** heat map. In the PLS-DA plot, circles with same colors represent replicates of metabolic profiles for each cultivar. The colored ellipses indicate 95% confidence regions of metabolic profiles for each cultivar. In the VIP score plot, 15 out of 26 metabolites in total were shown, and the colored boxes on the right indicate the relative abundance of the corresponding metabolite in each cultivar. Red represents higher relative abundance, whereas green and blue represent lower relative abundance in the VIP score plot and the heat map, respectively.

**Anti-inflammatory profiles of black walnut.** The anti-inflammatory profiles of the kernel extracts from 10 black walnut cultivars were characterized using data acquired from liquid chromatography–high-resolution MS (LC-HRMS). The UHPLC-HRMS data processed with XCMS Online provided 650 and 420 significant features in positive and negative modes, respectively, which were further annotated using METLIN metabolite database. This resulted in the identification of 26 substances with known anti-inflammatory activities. These metabolites included flavanols, hydroxybenzoic acids, and ellagitannins (**Supplementary Table 1**). The PLS-DA score plot showed significant differences in anti-inflammatory profiles among 10 black walnut cultivars (**Figure 1A**). In the score plot, Sparrow and Schessler, Mystry and Sparks, and Daniel and Davidson were distributed separately on quadrants I, II, and IV, respectively, whereas other four cultivars including Kwik Krop, Jackson, Hay, and Surprise roughly clustered together in the intersection of quadrants I and IV. Five cultivars (Daniel, Mystry, Sparks, Sparrow, and Surprise) were chosen as representative examples for further examined for their ability to alter cytokine production using human promonocytic cell line U-937 because they were selected from different groups distributed in four distinct quadrants (I, II, IV, or the intersection of I and IV). This approach was utilized to select cultivars that capture the most potentially diverse set of anti-inflammatory compounds.

Each black walnut cultivar contained several potential anti-inflammatory compounds, and the relative concentrations of many compounds were distinct within specific cultivars (**Figures 1B, C**). Sparks and Mystry contained the highest relative abundance of gallic acid and four ellagitannins including tellimagrandin I isomer 1 and 3, punicalin isomer 1, and pedunculagin isomer 1, while cinnamic acid and quercetin neohesperidoside were relatively dominant in Daniel and Davidson. The highest relative abundance of ellagic acid was found in Surprise, and this cultivar also contained procyanidin dimer, quercetin neohesperidoside, and pedunculagin isomer 1 as major components. Kwik Krop contained the highest relative abundance of myricetin hexoside, procyanidin dimer, punicalin isomer 3, and dicaffeocylquinic acid isomer 2. Schessler and Sparrow also contained a variety of bioactive compounds. Quercetin glucoside, myricetin, and pedunculagin isomer 3 were presented dominantly in Sparrow, while vanillic acid and dicaffeoylquinic acid isomer 3 were major compounds in Schessler.

**Figure 2:**
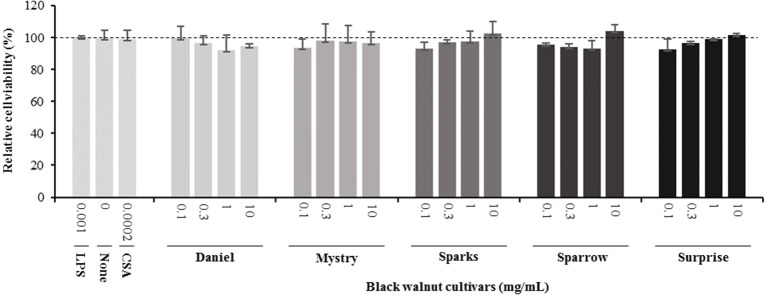
Effect of lipopolysaccharide (LPS), an immune suppressor drug (cyclosporin A, CSA), and black walnut extracts on the viability of PMA-differentiated U-937 cells. None: PMA-differentiated cells in the absence of black walnut extract and LPS. Mean ± SEM.

**Cell viability assays.** The cell viability assays were performed to address possible cytotoxic effects of the black walnut cultivars. A reduction in MTT absorbance could point to a decrease in cell viability, a decrease in mitochondrial activity, and/or cell loss, resulting in a concomitant decrease in cytokine secretion. MTT viability assays were performed at the same time cell supernatants were collected. CSA, a potent immunosuppressive agent capable of inhibiting cytokine production and release, was included in the experiments as a positive control. Black walnut extracts were resuspended in DMSO, and all experimental groups contained up to 0.1% DMSO. Control groups without extracts were supplemented with 0.1% DMSO to account for any adverse effects of the DMSO vehicle. The results indicated that the viability was not significantly different in any of the groups analyzed (**Figure 2**). These groups include no treatment (none, no LPS, and no black walnut extracts, with DMSO), LPS control cultures (LPS: LPS and DMSO, no extract), treated cells (Daniel, Mystry, Sparks, Sparrow, and Surprise: extracts, DMSO, and LPS), and CSA (CSA: CSA, DMSO, and LPS). Since the extracts of all five cultivars and CSA at all the concentrations tested were nontoxic to the PMA-differentiated U-937 cells, they were selected for examining their effect on cytokine production.

**Figure 3:**
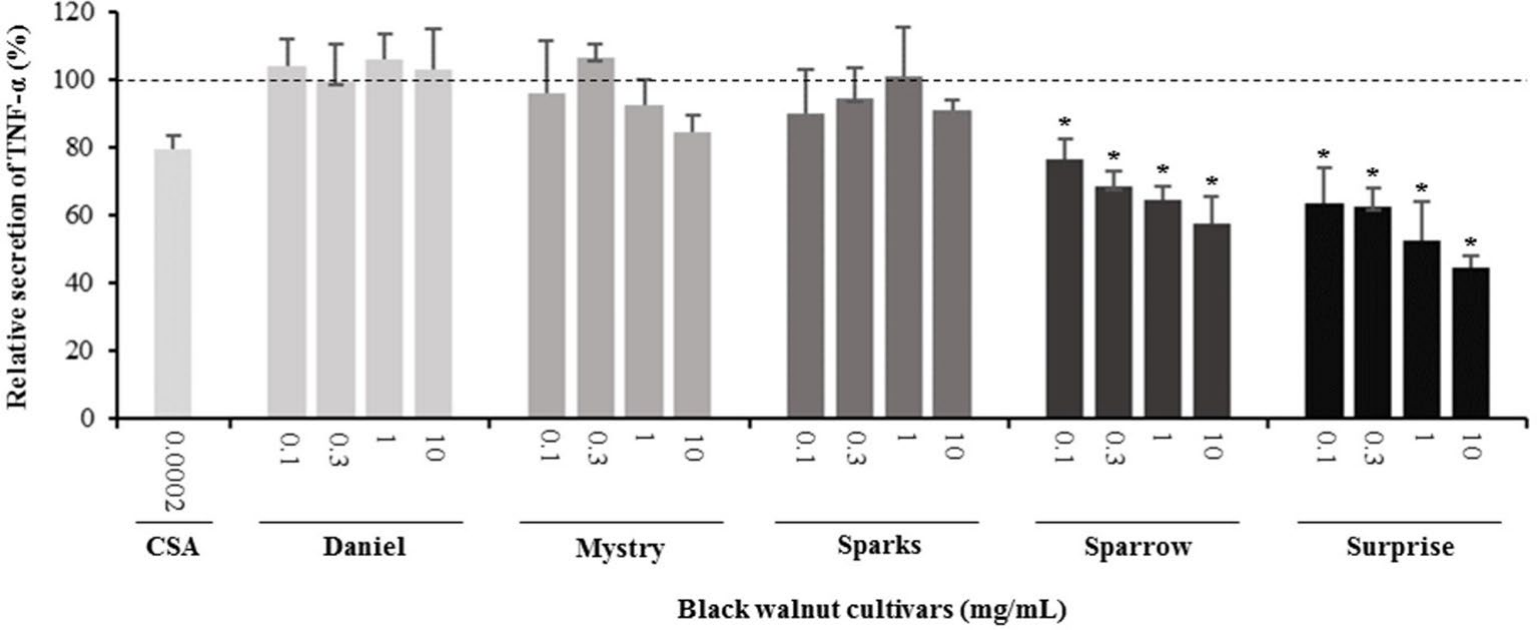
Effect of black walnut extracts on the secretion of TNF-α by PMA-differentiated, LPS-stimulated U937 cells. Cyclosporin A (CSA) was used as a positive control. (*) Significant decrease (*p* < 0.01) compared to PMA-differentiated, LPS-stimulated U937 cells in the absence of extract. Mean ± SEM.

**Figure 4:**
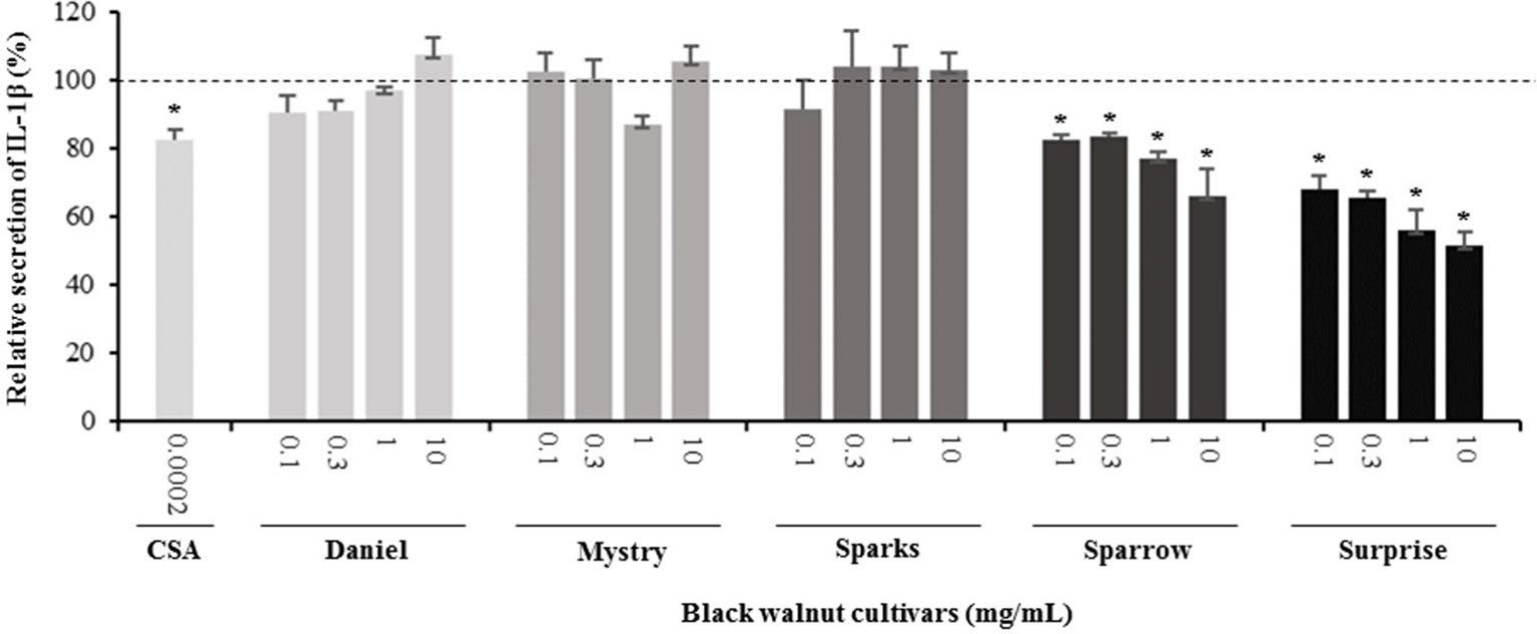
Effect of black walnut extracts on the secretion of IL-1β by PMA-differentiated, LPS-stimulated U937 cells. Cyclosporin A (CSA) was used as a positive control. (*) Significant decrease (*p* < 0.01) compared to PMA-differentiated, LPS-stimulated U937 cells in the absence of extracts. Mean ± SEM.

**Figure 5:**
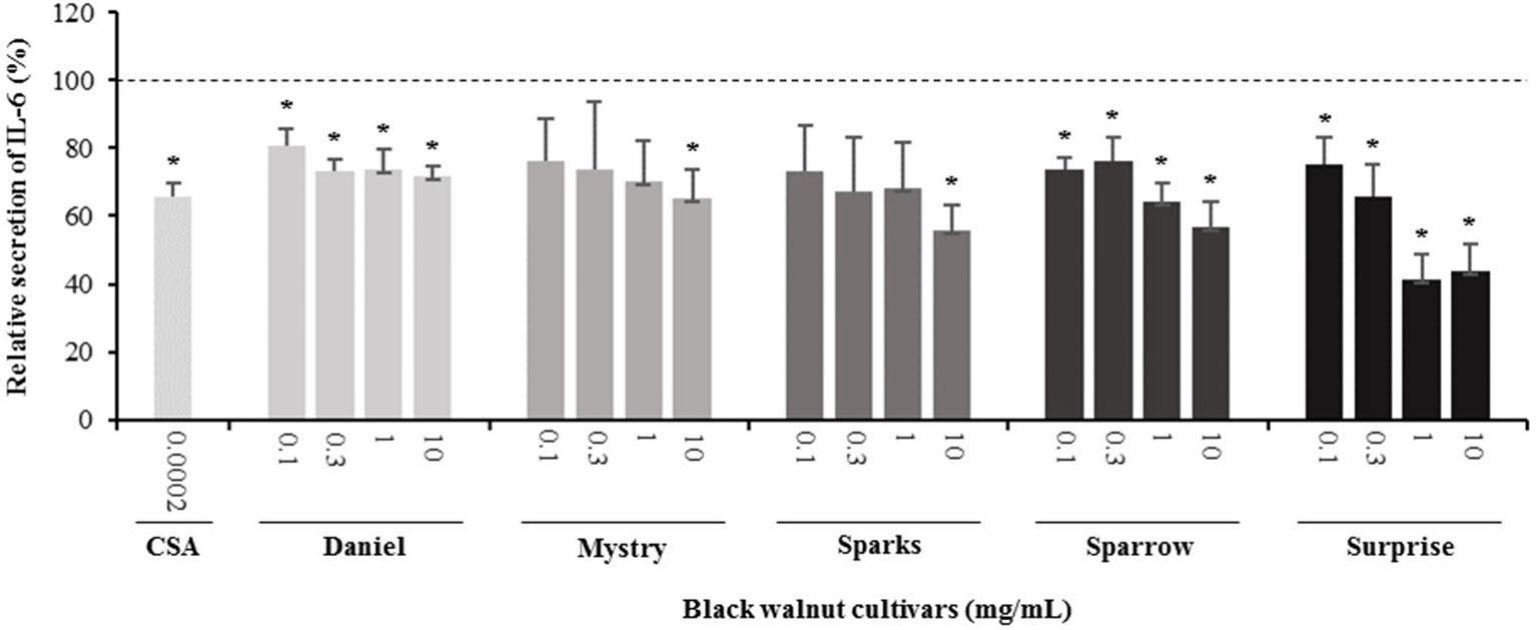
Effect of black walnut extracts on the secretion of IL-6 by PMA-differentiated, LPS-stimulated U937 cells. Cyclosporin A (CSA) was used as a positive control. (*) Significant decrease (*p* < 0.01) compared to PMA-differentiated, LPS-stimulated U937 cells in the absence of extract. Mean ± SEM.

**Figure 6:**
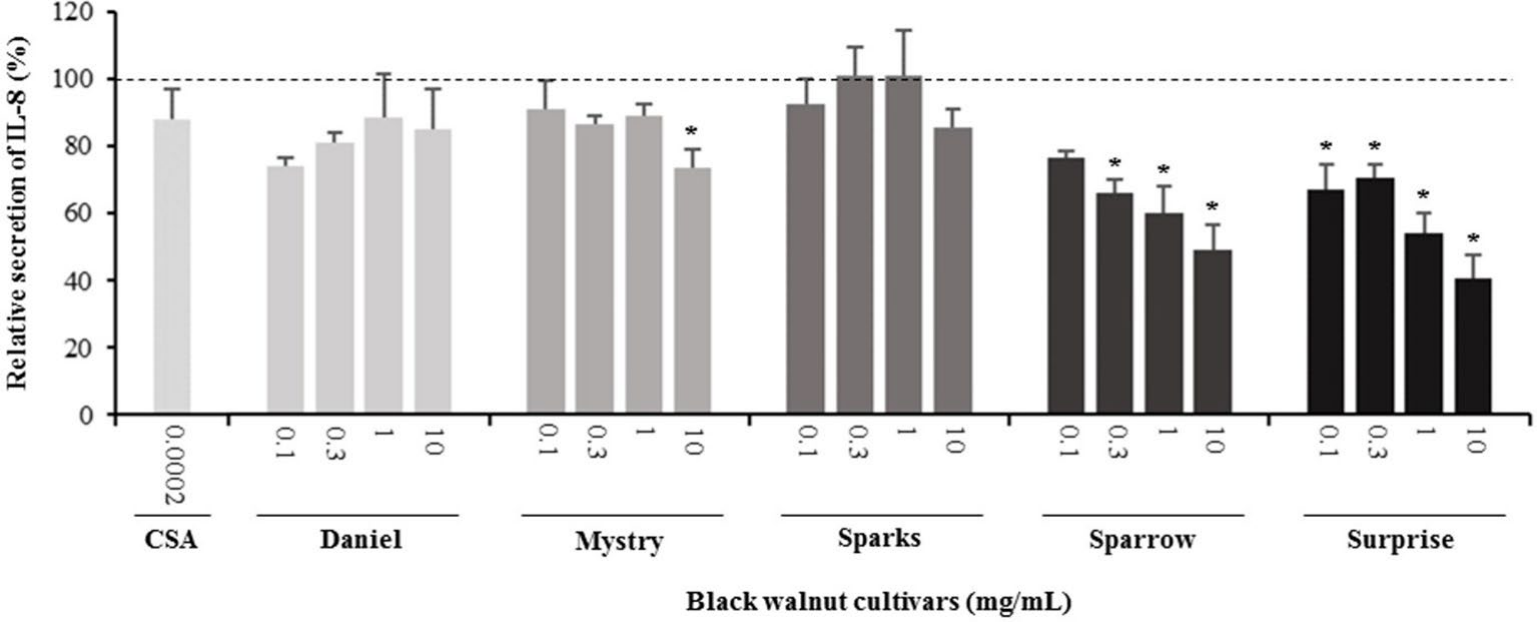
Effect of black walnut extracts on the secretion of IL-8 by PMA-differentiated, LPS-stimulated U937 cells. Cyclosporin A (CSA) was used as a positive control. (*) Significant decrease (*p* < 0.01) compared to PMA-differentiated, LPS-stimulated U937 cells in the absence of extract. Mean ± SEM.

**Figure 7:**
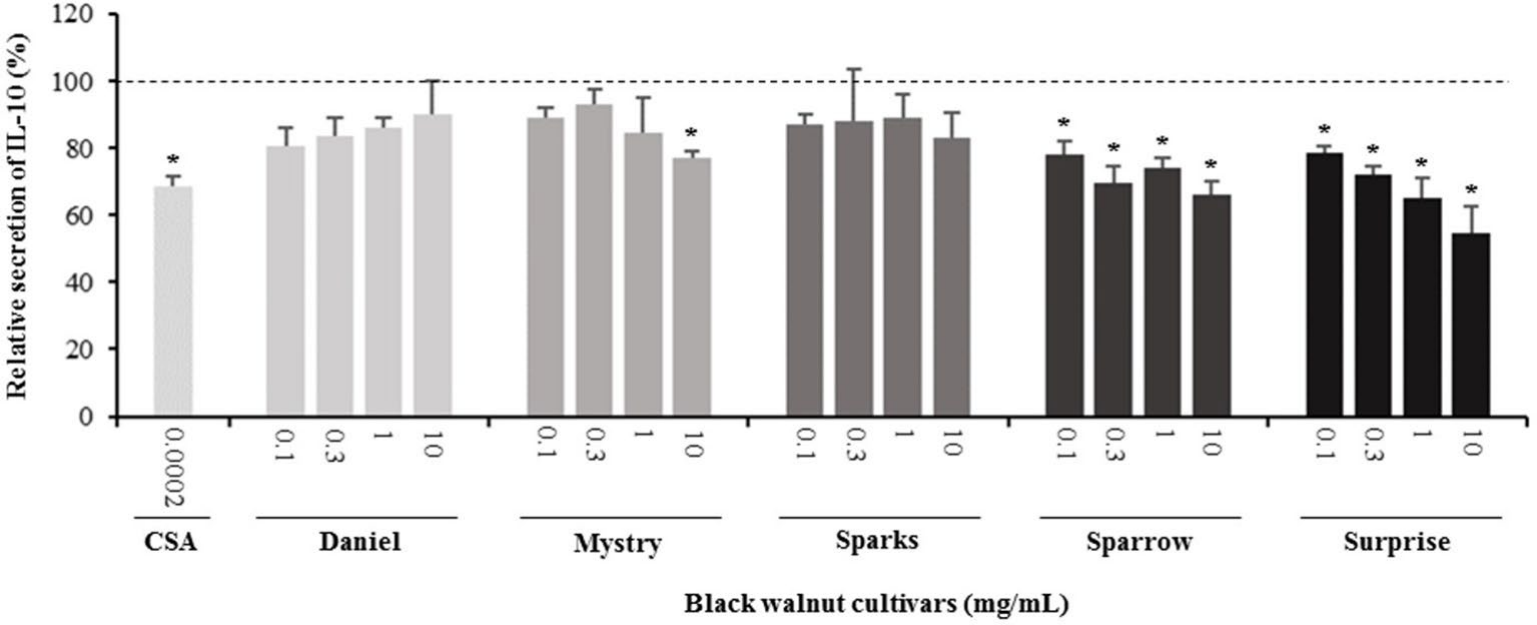
Effect of black walnut extracts on the secretion of IL-10 by PMA-differentiated, LPS-stimulated U937 cells. Cyclosporin A (CSA) was used as a positive control. (*) Significant decrease (*p* < 0.01) compared to PMA-differentiated, LPS-stimulated U937 cells in the absence of extract. Mean ± SEM.

**Figure 8:**
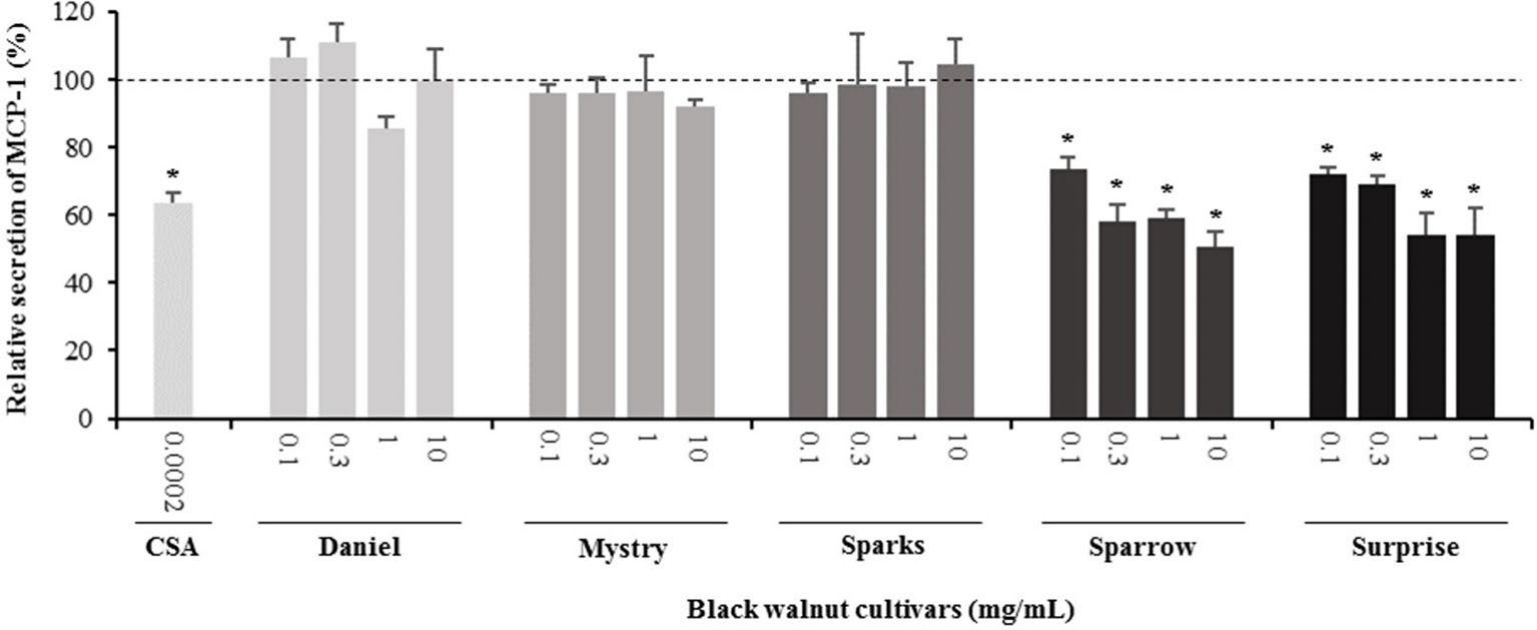
Effect of black walnut extracts on the secretion of MCP-1 by PMA-differentiated, LPS-stimulated U937 cells. Cyclosporin A (CSA) was used as a positive control. (*) Significant decrease (*p* < 0.01) compared to PMA-differentiated, LPS-stimulated U937 cells in the absence of extract. Mean ± SEM.

**Secretion of cytokines.** Secreted cytokine levels were quantified in PMA-differentiated, LPS-stimulated U-937 cells after pretreatment with five representative cultivars at four concentrations. A predefined human inflammation bead-based multiplex kit was chosen because it could be used to detect multiple cytokines simultaneously from the same sample with a small amount of the sample. The panel was comprised of 13 cytokines involved in various aspects of the inflammatory process, and many of these were studied previously in the U-937 model system. Of the six cytokines initially detected in the supernatants, five (TNF-α, IL-1β, IL-6, IL-8, and MCP-1) are proinflammatory and one (IL-10) is anti-inflammatory. Subsequent experiments focusing on these cytokines showed that secretion by PMA-differentiated, LPS-stimulated U-937 cells was significantly and dose-dependently attenuated by pretreatment with extracts derived from Sparrow and Surprise compared to control cells (**Figures 3**–**8**). More specifically, cultures pretreated with Sparrow extracts at the concentrations of 0.1, 0.3, 1, and 10 mg/ml reduced the secretion of TNF-α by 23.9%, 31.7%, 35.5%, and 42.7%, respectively; IL-1β by 17.7%, 16.4, 23.1, and 34.1%, respectively; IL-6 by 26.3%, 23.7%, 35.6%, and 43.3%, respectively; IL-8 by 23.4%, 34.1%, 39.8%, and 51.1%, respectively; IL-10 by 22.0%, 30.1%, 25.6%, and 33.9%, respectively; and MCP-1 by 26.5%, 41.9%, 40.8%, and 49.1%, respectively (**Figures 3**–**8**). Similarly, at the concentrations of 0.1, 0.3, 1, and 10 mg/ml, cultures pretreated with Surprise extracts also reduced the secretion of TNF-α by 36.4%, 37.6%, 47.8%, and 55.7%, respectively; IL-1β by 32.1%, 34.8%, 44.0%, and 48.7%, respectively; IL-6 by 24.9%, 34.3%, 58.9%, and 56.3%, respectively; IL-8 by 33.1%, 29.3%, 45.9%, and 59.3%, respectively; IL-10 by 21.2%, 27.8%, 34.9%, and 45.5%, respectively; and MCP-1 by 27.8%, 30.7%, 45.6%, and 46.0%, respectively (**Figures 3**–**8**).

Minor to no effects were observed in IL-1β, TNF-α, and MCP-1 cytokine levels following pretreatment with Daniel, Mystry, and Sparks at all four concentrations tested. The secretion of IL-6 was significantly inhibited by extracts derived from Daniel (≥0.1 mg/ml), Mystry (10 mg/ml), and Spark (10 mg/ml). More specifically, the extracts of Daniel at the concentrations of 0.1, 1, 0.3, and 10 mg/ml, Mystry and Sparks at a concentration of 10 mg/ml significantly reduced the secretion of IL-6 by 19.5%, 26.7%, 26.5%, 28.5%, 34.9%, and 44.1%, respectively (**Figure 5**). In addition, the extract of Mystry at a concentration of 10 mg/ml reduced the secretion of IL-8 (26.3%) and IL-10 (22.8%) by PMA-differentiated, LPS-stimulated U-937 cells (**Figures 6** and **7**).

In order to gain insight into potential mechanism(s) of action, parallel experiments whereby LPS was added 2 h before exposure to extracts were conducted. These experiments focused on Sparrow or Surprise cultivars because of their strong inhibitory effect on cytokine secretion among examined cultivars when the extracts were added before the addition of LPS. In these experiments, the cell stimulation and cytokine secretion were initiated before the extracts were added. The results showed that Sparrow and Surprise extracts had no inhibitory effect on the secretion of the six cytokines (TNF-α, IL-1β, IL-6, IL-8, IL-10, and MCP-1) when LPS was added before the extracts (data not shown).

## Discussion

The results of this study demonstrated for the first time that black walnut possesses compounds that exert an inhibitory effect on the secretion of six measured cytokines (TNF-α, IL-1β, IL-6, IL-8, IL-10, and MCP-1) induced by a human promonocytic cell line differentiated with PMA and stimulated with LPS. Cytokines play vital roles in mediating pathological responses in RA. TNF, IL-1, and IL-6 are pivotal cytokines in regulating innate and adaptive immune responses associated with the disease onset and persistence, and TNF and IL-1 are signature innate cytokines in RA. TNF is functionally central to RA pathophysiology and is involved in leukocyte activation, adhesion, and migration, in endothelial-cell adhesion molecule expression, and in stromal-cell, chondrocyte, and osteoclast activation (McInnes et al., 2016); IL-1 plays a role in destruction of bone at inflammatory joint sites, such as in RA, *via* activation, differentiation, and survival of osteoclasts (Kim et al., 2009), and it also mediates secretion of cytokines from synovial fibroblasts and monocytes and induces endothelial-cell adhesion molecule expression (McInnes and Schett, 2007). IL-6 is a key player in innate and adaptive immune responses, including proliferation, differentiation, cytotoxic T cell activity, antibody production, generation of blood cells and platelets, hepatic acute-phase responses, and neuroendocrine effects (McInnes and Schett, 2007). The two proinflammatory cytokines, TNF and IL-6, are therapeutic targets for the treatment of RA (Smolen and Aletaha, 2015), whereas inhibition of IL-1 using biological therapies has not been effective in preventing RA (McInnes et al., 2016). In the present study, we showed that black walnut cultivars Sparrow and Surprise significantly and dose-dependently reduced the secretion of three proinflammatory cytokines (TNF-α, IL-1β, and IL-6) in the U-937 model system. Additionally, other cultivars also had an inhibitory effect on IL-6 secretion. Our findings suggest that the extracts from certain black walnut cultivars, such as Sparrow and Surprise, might be promising biological agents for future investigation on the prevention of inflammatory diseases, such as RA in animal model studies.

Our results also revealed the inhibitory effects of black walnut extracts on three other cytokines/chemokines (MCP-1, IL-8, and IL-10) in the U-937 model system. MCP-1 and IL-8, members of the CC and CXC chemokine family, respectively, play important roles in acute inflammation. The expression of MCP-1 regulates migration and infiltration of monocytes, memory T cells, and dendritic cells (Carr et al., 1994, Deshmane et al., 2009), whereas IL-8 recruits neutrophils and other granulocytes to the site of infection and stimulates phagocytosis (Harada et al., 1994). IL-10 is a potent immune-modulatory cytokine with broad anti-inflammatory properties (O’garra et al., 2008) and is produced by a variety of cells including B cells, Th1, Th2, Th17, T reg, CD8+ T cells, and myeloid cells (Saraiva and O’garra, 2010). During inflammation, IL-10 can be produced by regulatory B cells and inhibit the production of TNF (Smallie et al., 2010) as well as inhibit the infiltration and activation of neutrophils in the synovial tissue (Bober et al., 2000). Interestingly, Sparrow and Surprise exhibited inhibitory effects not only on MCP-1 and IL-8 but also on IL-10. Black walnut extracts contain many possible anti-inflammatory metabolites (**Figure 1**) that might exert the inhibitory effect on the production of IL-8 and MCP-1 (**Figure 1**) as well as other unidentified metabolites. Some anti-inflammatory compounds might have broad cytokine suppressive effect that might inhibit the production of IL-10 in the U-937 model system.

Our results indicated a rich source of possible anti-inflammatory bioactive compounds in black walnut. These compounds were tentatively identified through a metabolomics analysis (**Figure 1**), and many of them, including gallic acid, vanillic acid, ellagic acid, quercetin, quercetin 3-*O*-glucoside, naringin, and cinnamic acid, have been confirmed and quantified in black walnut kernels using LC-MS/MS analysis with authentic standards (Vu et al., 2018). The presence of multiple compounds in certain cultivars raises the possibility of synergistic effects on cytokine secretion. We previously identified multiple bioactive compounds (e.g., glansreginin A, azelaic acid, and quercetin) in black walnut that are responsible for antibacterial activities against a Gram-positive bacterium (*S. aureus* RN6390) (Ho et al., 2018). Bioassay-guided fractionation was utilized to isolate and identify the bioactive compounds (based on MS/MS fragmentation data of ions) by a metabolomics approach using high-resolution MS/MS databases (e.g., METLIN, MassBank of North America, MetFrag). Future research will focus on purification, validation, and characterization of compounds responsible for cytokine suppressive activities in Surprise and Sparrow utilizing the same metabolomics strategy. The compounds in black walnut might be useful as natural anti-inflammatory agents to mitigate inflammation.

Interestingly, the inhibitory effect of Surprise and Sparrow cultivars was only observed when U-937 were pretreated with the extracts of these cultivars before the addition of LPS, whereas the suppressive activity did not occur when U-937 were stimulated with LPS before addition of the extract. These findings suggest the possibility that the extracts are inhibiting cell activation at the level of the receptor rather than affecting downstream signaling pathways. The observed decrease in cytokine levels in the pretreated experiments could result from the ability of the extract to interfere with the interaction between LPS and its receptor TLR4 and/or coreceptor CD14, or from the direct binding of the extract to the LPS receptor, thereby blocking cell activation. In other words, the extracts might function by preventing LPS from activating U-937 cells. Importantly, TLR4 receptors are expressed in U-937 cells, and receptor activation leads to a dose-dependent induction of certain inflammatory markers (Vogel et al., 2012).When U-937 were stimulated with LPS before the addition of the extracts, LPS might bind to its receptor and initiate the signaling cascade before the addition of the extracts. Additionally, the active compounds may be effective against other receptors and/or affect intracellular signaling pathways. The mechanism of action is not fully understood at this point. Future experiments will be required to elucidate how the active compounds are carrying out their effect.

We also documented that cytokine suppressive activities of black walnut were variable among tested cultivars. Two cultivars (Sparrow and Surprise) exhibited inhibitory effect on the secretion of six detected cytokines induced by U-937 differentiated with PMA and stimulated with LPS, while other cultivars (Daniel, Mystry, and Sparks) reduced the secretion of IL-6 alone. Mystry also reduced the secretion of IL-10 and MCP-1, but no inhibitory effect of Mystry on TNF-α, IL-1β, and IL-8 was observed. Differences in the suppressive properties of black walnut are likely due to the differences in the composition and proportions of different compounds among these cultivars (**Figure 1A**). In our previous studies, we also found differences in antibacterial properties among black walnut cultivars (Ho et al., 2018). In fact, Surprise and Mystry exhibited the strongest antibacterial capacities against *S. aureus* among 22 tested cultivars, while Daniel, Davidson, Hay, Jackson, Kwik Krop, Schessler, Sparks, and Sparrow cultivars showed no effect on the inhibition of *S. aureus*. Health-promoting characteristics of black walnut (e.g., anti-inflammatory and antibacterial capacities) could potentially be used to select traits to improve nut quality. Over 700 black walnut cultivars have been recorded, and many of them were selected for nut production in the past century (Williams, 1990). Current nut production criteria include yield, percent kernel, leafing date, flowering dates, growth habit, disease resistance, precocity, productivity, and shelling quality (Reid et al., 2004).

## Conclusion

We demonstrated cytokine suppressive properties of black walnut extracts using the human promonocytic cell line U-937. Black walnut kernels contain a wealth of bioactive metabolites putatively identified through a metabolomics approach. The five cultivars (Daniel, Mystry, Sparks, Sparrow, and Surprise) tested showed differences in their ability to inhibit the secretion of six cytokines/chemokines (TNF-α, IL-1β, IL-6, IL-8, IL-10, and MCP-1). Sparrow and Surprise showed the strongest inhibitory effects on the secretion of all measured cytokines. Mystry reduced the secretion of IL-6, IL-10, and MCP-1, while Daniel and Sparks only reduced the production of IL-6. Our findings reveal that certain black walnut cultivars may represent promising preventive agents for inflammatory diseases. In addition, this could potentially have an application in the cosmeceutical field related to inflammatory skin disorders.

## Data availability

All datasets generated for this study are included in the manuscript and the **Supplementary files**.

## Author Contributions

C–HL contributed to conception of the study. K–VH wrote the first draft of the manuscript. K–VH and KS designed the experiments and performed the analyses. K–VH, KS, and DV performed the experiments. KS, ZL, LS, MC, and C–HL provided materials required for the experiments. All authors edited and approved the final version of the manuscript.

## Conflict of Interest Statement

The authors declare that the research was conducted in the absence of any commercial or financial relationships that could be construed as a potential conflict of interest.
