# Supplementary material for: Black Walnut (Juglans nigra) Extracts Inhibit Proinflammatory Cytokine Production From Lipopolysaccharide-Stimulated Human Promonocytic Cell Line U-937
Source: Front Pharmacol. 2019 Sep 19;10:1059. doi: 10.3389/fphar.2019.01059 (PMC6761373; doi:10.3389/fphar.2019.01059)
Supplement: Supplementary file 1 [file Table_1.docx]

Supplementary Material

**Black walnut (*Juglans nigra*) extracts inhibit pro-inflammatory cytokine production from LPS-stimulated human pro-monocytic cell line U-937**

Khanh-Van Ho^1,2^, Kathy L. Schreiber^3^, Danh C. Vu^1^, Susan M. Rottinghaus^3^, Daniel E. Jackson^3^, Charles R. Brown^4^, Zhentian Lei^5,6^, Lloyd W. Sumner^5,6^, Mark V. Coggeshall^7^, and Chung-Ho Lin^1*^

^1^ Center for Agroforestry, School of Natural Resources, University of Missouri, Columbia, MO 65211, USA

^2^ Department of Food Technology, Can Tho University, Can Tho 90000, Vietnam

^3^ Cell and Immunobiology Core, University of Missouri, Columbia, MO 65211, USA

^4^ Department of Veterinary Pathobiology, University of Missouri, Columbia, MO 65211, USA

^5^ Metabolomics Center, University of Missouri, Columbia, MO 65211, USA

^6^ Department of Biochemistry, Bond Life Sciences Center, University of Missouri, Columbia, MO 65211

^7^ United States Northern Research Station, USDA-Forest Service, West Lafayette, IN 47907, USA

* Corresponding author: Chung-Ho Lin, Linchu@missouri.edu

**Supplementary Table 1**. Phenolic compounds known to produce anti-inflammatory activity and putatively identified in the methanolic extracts of black walnut kernels via a metabolomics approach.

| Feature  No. | Retention time (min) | Theoretical mass | Exact  mass | Δm  (ppm) | Adducts | Formula | Putatively identified compound | Anti-inflammatory activity |
| --- | --- | --- | --- | --- | --- | --- | --- | --- |
| 1 | 0.59 | 787.0988 | 787.0995 | 0.89 | [M + H]^+^ | C_34_H_26_O_22_ | Tellimagrandin I isomer 1  (di-HHDP glucose isomer) | Kiss and Piwowarski (2018) |
| 2 | 1.10 | 785.0832 | 785.0846 | 1.78 | [M + H]^+^ | C_34_H_24_O_22_ | Pedunculagin isomer 1  (bis-HHDP-glucose) | Lee et al. (2010) |
| 3 | 1.23 | 577.1352 | 577.1359 | 1.21 | [M – H]^–^ | C_30_H_26_O_12_ | Procyanidin dimer | Xing et al. (2015) |
| 4 | 1.87 | 787.0988 | 787.0976 | -1.52 | [M + H]^+^ | C_34_H_26_O_22_ | Tellimagrandin I isomer 2 | Kiss and Piwowarski (2018) |
| 5 | 2.23 | 169.0131 | 169.0129 | -1.18 | [M – H]^–^ | C_7_H_6_O_5_ | Gallic acid | Kim et al. (2005) |
| 6 | 2.35 | 499.1234 | 499.1210 | -4.81 | [M + H – H_2_O]^+^ | C_25_H_24_O_12_ | Dicaffeoylquinic acid isomer 1 | Han et al. (2007) |
| 7 | 2.61 | 787.0988 | 787.0995 | 0.89 | [M + H]^+^ | C_34_H_26_O_22_ | Tellimagrandin I isomer 3 | Kiss and Piwowarski (2018) |
| 8 | 2.74 | 463.0871 | 463.0898 | 5.83 | [M + H – H_2_O]^+^ | C_21_H_20_O_13_ | Myricetin hexoside isomer | Wang et al. (2010) |
| 9 | 3.43 | 169.0349 | 169.0490 | -2.96 | [M + H]^+^ | C_8_H_8_O_4_ | Vanillic acid | Miles et al. (2005) |
| 10 | 3.44 | 785.0832 | 785.0836 | 0.51 | [M + H]^+^ | C_34_H_24_O_22_ | Pedunculagin isomer 2 | Lee et al. (2010) |
| 11 | 3.45 | 783.0675 | 783.0641 | -4.34 | [M + H]^+^ | C_34_H_22_O_22_ | Punicalin isomer 1 | Lee et al. (2010) |
| 12 | 4.37 | 303.0135 | 303.0138 | 0.99 | [M + H]^+^ | C_14_H_6_O_8_ | Ellagic acid | Umesalma and Sudhandiran (2010) |
| 13 | 4.77 | 319.0448 | 319.0455 | 2.19 | [M + H]^+^ | C_15_H_10_O_8_ | Myricetin | Wang et al. (2010) |
| 14 | 5.14 | 463.0882 | 463.0867 | -3.23 | [M – H]^–^ | C_21_H_20_O_12_ | Quercetin 3-*O*-glucoside | Rogerio et al. (2007) |
| 15 | 5.30 | 441.0827 | 441.0790 | -8.39 | [M – H]^–^ | C_22_H_18_O_10_ | (-)-Epicatechin 3-*O*-gallate | Kürbitz et al. (2011) |
| 16 | 6.42 | 479.1184 | 479.1191 | 1.46 | [M + H]^+^ | C_22_H_22_O_12_ | Isorhamnetin hexoside isomer | Abdallah and Esmat (2017) |
| 17 | 6.59 | 499.1234 | 499.1199 | -7.21 | [M + H – H_2_O]^+^ | C_25_H_24_O_12_ | Dicaffeoylquinic acid isomer 2 | Han et al. (2007) |
| 18 | 8.10 | 303.0499 | 303.0498 | -0.32 | [M + H]^+^ | C_15_H_10_O_7_ | Quercetin | Rogerio et al. (2007) |
| 19 | 10.68 | 783.0675 | 783.0678 | 0.38 | [M + H]^+^ | C_34_H_22_O_22_ | Punicalin isomer 2 | Lee et al. (2010) |
| 20 | 19.21 | 581.1865 | 581.1843 | -3.78 | [M + H]^+^ | C_27_H_32_O_14_ | Naringin | Golechha et al. (2011) |
| 21 | 20.84 | 785.0832 | 785.0823 | -1.15 | [M + H]^+^ | C_34_H_24_O_22_ | Pedunculagin isomer 3 | Lee et al. (2010) |
| 22 | 25.77 | 149.0597 | 149.0599 | 1.34 | [M + H]^+^ | C_9_H_8_O_2_ | Cinnamic acid | Song et al. (2013) |
| 23 | 28.31 | 441.0816 | 441.0817 | 0.22 | [M + H – H_2_O]^+^ | C_22_H_18_O_11_ | (-)-Epigallocatechin gallate | Cavet et al. (2011) |
| 24 | 32.77 | 593.1501 | 593.1532 | 5.23 | [M + H – H_2_O]^+^ | C_27_H_30_O_16_ | Quercetin 3-*O*-neohesperidoside | da Silva et al. (2000) |
| 25 | 35.59 | 783.0675 | 783.0674 | -0.13 | [M + H]^+^ | C_34_H_22_O_22_ | Punicalin isomer 3 | Lee et al. (2010) |
| 26 | 39.79 | 299.0550 | 299.0557 | 2.34 | [M + H – H_2_O]^+^ | C_16_H_12_O_7_ | 3’-Methylquercetin | Jiang et al. (2006) |

**References**

Abdallah, H. M. and Esmat, A. (2017). "Antioxidant and anti-inflammatory activities of the major phenolics from *Zygophyllum simplex* L." J. Ethnopharmacol. 205: 51-56.

Cavet, M. E., Harrington, K. L., Vollmer, T. R., Ward, K. W. and Zhang, J.-Z. (2011). "Anti-inflammatory and anti-oxidative effects of the green tea polyphenol epigallocatechin gallate in human corneal epithelial cells." Mol. Vis. 17: 533.

da Silva, B. P., Bernardo, R. R. and Parente, J. P. (2000). "Flavonol glycosides from *Costus spicatus*." Phytochemistry 53(1): 87-92.

Golechha, M., Chaudhry, U., Bhatia, J., Saluja, D. and Arya, D. S. (2011). "Naringin protects against kainic acid-induced status epilepticus in rats: evidence for an antioxidant, anti-inflammatory and neuroprotective intervention." Biol. Pharm. Bull. 34(3): 360-365.

Han, T., Li, H.-L., Zhang, Q.-Y., Han, P., Zheng, H.-C., Rahman, K., et al. (2007). "Bioactivity-guided fractionation for anti-inflammatory and analgesic properties and constituents of *Xanthium strumarium* L." Phytomedicine 14(12): 825-829.

Jiang, J.-S., Shih, C.-M., Wang, S.-H., Chen, T.-T., Lin, C.-N. and Ko, W.-C. (2006). "Mechanisms of suppression of nitric oxide production by 3-O-methylquercetin in RAW 264.7 cells." J. Ethnopharmacol. 103(2): 281-287.

Kim, S.-H., Jun, C.-D., Suk, K., Choi, B.-J., Lim, H., Park, S., et al. (2005). "Gallic acid inhibits histamine release and pro-inflammatory cytokine production in mast cells." Toxicol. Sci. 91(1): 123-131.

Kiss, A. K. and Piwowarski, J. P. (2018). "Ellagitannins, gallotannins and their metabolites-the contribution to the anti-inflammatory effect of food products and medicinal plants." Curr. Med. Chem. 25(37): 4946-4967.

Kürbitz, C., Heise, D., Redmer, T., Goumas, F., Arlt, A., Lemke, J., et al. (2011). "Epicatechin gallate and catechin gallate are superior to epigallocatechin gallate in growth suppression and anti‐inflammatory activities in pancreatic tumor cells." Cancer Sci. 102(4): 728-734.

Lee, C.-J., Chen, L.-G., Liang, W.-L. and Wang, C.-C. (2010). "Anti-inflammatory effects of *Punica granatum* Linne invitro and in vivo." Food Chem. 118(2): 315-322.

Miles, E. A., Zoubouli, P. and Calder, P. C. (2005). "Differential anti-inflammatory effects of phenolic compounds from extra virgin olive oil identified in human whole blood cultures." Nutrition 21(3): 389-394.

Rogerio, A. P., Kanashiro, A., Fontanari, C., Da Silva, E. V. G., Lucisano-Valim, Y. M., Soares, E. G., et al. (2007). "Anti-inflammatory activity of quercetin and isoquercitrin in experimental murine allergic asthma." Inflamm. Res. 56(10): 402-408.

Song, F., Li, H., Sun, J. and Wang, S. (2013). "Protective effects of cinnamic acid and cinnamic aldehyde on isoproterenol-induced acute myocardial ischemia in rats." J. Ethnopharmacol. 150(1): 125-130.

Umesalma, S. and Sudhandiran, G. J. B. (2010). "Differential inhibitory effects of the polyphenol ellagic acid on inflammatory mediators NF‐κB, iNOS, COX‐2, TNF‐α, and IL‐6 in 1, 2‐dimethylhydrazine‐induced rat colon carcinogenesis." Basic Clin. Pharmacol. Toxicol. 107(2): 650-655.

Wang, S.-J., Tong, Y., Lu, S., Yang, R., Liao, X., Xu, Y.-F., et al. (2010). "Anti-inflammatory activity of myricetin isolated from *Myrica rubra* Sieb. et Zucc. leaves." Planta Medica 76(14): 1492-1496.

Xing, J., Li, R., Li, N., Zhang, J., Li, Y., Gong, P., et al. (2015). "Anti-inflammatory effect of procyanidin B1 on LPS-treated THP1 cells via interaction with the TLR4–MD-2 heterodimer and p38 MAPK and NF-κB signaling." Mol. Cell. Biochem. 407(1-2): 89-95.
